# Effect of Routine Varicella Immunization on the Epidemiology and Immunogenicity of Varicella and Shingles

**DOI:** 10.3390/v14030588

**Published:** 2022-03-12

**Authors:** Naruhito Otani, Masayuki Shima, Takuma Yamamoto, Toshiomi Okuno

**Affiliations:** 1 Department of Public Health, Hyogo College of Medicine, Nishinomiya 663-8501, Japan; shima-m@hyo-med.ac.jp; 2 Department of Legal Medicine, Hyogo College of Medicine, Nishinomiya 663-8501, Japan; tk-yamamoto@hyo-med.ac.jp; 3 Department of Microbiology, Hyogo College of Medicine, Nishinomiya 663-8501, Japan; tmokuno@hyo-med.ac.jp

**Keywords:** herpes zoster virus, varicella, herpes zoster, vaccine, vaccination, humoral immunity, cell-mediated immunity

## Abstract

Varicella-zoster virus (VZV) causes varicella as a primary infection and remains latent in the ganglia until it becomes reactivated to cause herpes zoster. Individuals with varicella develop adaptive humoral and cell-mediated immunity. Compromised cell-mediated immunity is thought to contribute to the development of herpes zoster. Recent evidence suggests that changes in the epidemiology of varicella have affected the epidemiology of herpes zoster. The incidence of herpes zoster is higher in older adults; thus, the herpes zoster vaccine is recommended for older adults. However, the incidence of herpes zoster is expected to rise among younger individuals; hence, vaccination with the varicella vaccine should also be considered in younger adults. In order to determine the need for vaccination in different populations, it is important to establish methods to accurately assess the activity of cell-mediated immunity and humoral immunity.

## 1. Introduction

The first varicella vaccine was developed in Japan in 1974 by Takahashi et al. [1] and was subsequently approved for use in high-risk children in Europe in 1984. In Japan, it was approved in 1986 and became commercially available in 1987. In the United States, the US Food and Drug Administration (FDA) approved the first varicella vaccine in 1995 [2]. Varicella vaccines have subsequently become part of routine immunization programs in several countries.

Vaccination has dramatically changed the epidemiology of varicella in countries with routine immunization programs. In Japan, routine immunization for varicella has been shown to affect the epidemiology of herpes zoster [3]. Immunologically, a compromised immune system is considered a risk factor for herpes zoster [4].

Varicella-zoster virus (VZV) causes varicella as a primary infection. Latent VZV is reactivated to cause herpes zoster. The pathogenesis of the disease has been studied using the mousepox [5] and SCID-hu mouse [6] models, although its exact mechanism remains unclear. The role of humoral immunity in VZV infection has been well-described, as antibodies are known to be involved in the development of varicella. However, little is known about the role of cell-mediated immunity (CMI) against VZV, which is thought to play an important role in the development of herpes zoster [7]. Thus, there is a need for novel testing methods to accurately assess the activity of CMI. To date, few studies have examined the level of CMI against VZV [8,9,10]. Intradermal testing is the only method that is currently being used to assess CMI in clinical settings.

## 2. Epidemiology of Varicella Following Routine Immunization

### 2.1. United States

In the United States, the annual incidence of varicella was approximately 4,000,000 (15 per 1000 population) in the 1980s and 1990s [2,11]. The FDA approved the varicella vaccine in 1995, and routine immunization against varicella was implemented in 1996. At that time, one dose of vaccine was recommended for children aged 12 months to 12 years, and two doses of vaccine, administered 4–8 weeks apart, were recommended for children aged 13 years and over [2,12]. However, studies subsequently demonstrated that a single dose was ineffective at preventing varicella because of a high incidence of breakthrough infections [2]. Thus, a new set of recommendations was developed in 2005 that recommended that children aged 12 months to 12 years receive two doses of vaccine: the first at the age of 12–15 months, and the second at the age of 4–6 years [2]. This two-dose varicella vaccination policy is now accepted by the Advisory Committee on Immunization Practices (ACIP) as one of the criteria for the proof of varicella immunization [2].

Following the introduction of varicella vaccination, the incidence rate of varicella decreased by approximately 90% between 1995 and 2005, from 4.1 cases per 1000 population in 1995, to 0.4 cases per 1000 population in 2005 [13]. By 2005, the vaccination coverage among children aged 19–35 months reached 87.9% [14].

Following the 2005 recommendation of a two-dose varicella vaccination strategy, the incidence rate of varicella decreased by a further 83.6% from 25.4 cases per 100,000 population in 2005–2006, to 3.9 cases per 100,000 population in 2013–2014 [15]. By 2013–2014, 93.3% of the eligible population had received two doses of the vaccine [16].

The incidence of varicella in children aged 1–14 years decreased by approximately 90% from 1995 to 2005 following the introduction of the first varicella vaccination strategy [13]. Specifically, between 2005–2006 and 2013–2014, the incidence rate decreased by 58%, 89.3%, and 84.8% in children aged 1–4 years, 5–9 years, and 10–14 years, respectively [15]. The decrease was most apparent in children aged 5–9 years, probably because of the introduction of a second vaccination at the age of 4–6 years.

### 2.2. Japan

In Japan, as the varicella vaccine was not used for routine immunization after its approval in 1986, the incidence of varicella was approximately 1,000,000 cases annually, with most cases occurring in children [17,18]. Routine immunization with two doses of the vaccine was introduced in 2014. It is recommended that the second dose should be administered at least 3 months after the first dose, at the age of 12–36 months. Following the introduction of routine varicella immunization in 2014, the incidence rate of varicella decreased substantially in 2015 [19] and decreased by 78% in 2019 compared with the 2000–2011 average annual incidence rate [20]. There was a substantial decrease in the incidence of varicella in children, with those under the age of 4 years representing approximately 80% of all cases prior to the introduction of the routine immunization program and only 40% of all cases in 2017 [19]. The decrease in the incidence rate plateaued in 2018 and 2019 [21]. Data from sentinel surveillance sites (approximately 3000) showed that the incidence rate of varicella decreased from 81.4 cases/year in 2000 to 18 cases/year in 2019 [21]. The vaccination coverage in 2018 was reported to be approximately 70.3% [22]. The incidence rate decreased in 2020 and 2021, probably because of the effect of droplet infection control measures to prevent the spread of COVID-19, in addition to the effect of vaccination [23].

## 3. Epidemiology of Herpes Zoster

Herpes zoster is a common disease that results from the reactivation of latent VZV. In 1965, Hope-Simpson [24] reported that the annual incidence rate of herpes zoster was 3.4 cases per 1000 population in England. Herpes zoster affects approximately 1,000,000 individuals per year in the United States [25,26]. The annual incidence rate increases with age, from 5 in 1000 population in the 50–59-year age group to 11 in 1000 in individuals over 80 years of age [25,26]. In Japan, the Miyazaki study examined the incidence of herpes zoster from 1997 to 2006 and estimated an annual incidence rate of 4.15 cases per 1000 population [3]. Kawai et al. [27] reviewed various studies from different countries and estimated that the annual incidence rate of herpes zoster ranged from 3 to 5 cases per 1000 population.

Yih et al. [28] reported that the incidence of herpes zoster in Massachusetts in the United States increased from 1998 to 2003, while the incidence of varicella decreased because of an increase in varicella vaccine coverage. However, an age-specific increase in the incidence of herpes zoster was reported in the United States even before the varicella vaccination program was initiated [29]. Therefore, the increase in the incidence of herpes zoster in the United States may not be solely attributable to varicella vaccination.

The varicella vaccine (the Oka strain) is reported to be associated with a lower risk of herpes zoster than natural infection with wild-type VZV [30,31]. This is thought to be because vaccination with the Oka strain can reduce the viral load in the ganglia [32]. Therefore, childhood varicella vaccination may be important in terms of prevention of shingles in later life. However, the effective duration of childhood vaccination is unknown, and epidemiological studies are warranted to examine whether a booster vaccination is necessary.

McKay et al. [33] reported that the incidence of herpes zoster in immunocompromised adults ranged from 9 to 92 cases per 1000 population, with the highest incidence in individuals who had undergone hematopoietic stem cell transplantation, followed by those with hematological malignancies, solid organ transplantation, and solid tumor malignancies; the incidence was lowest in individuals with HIV infection. Even in the population with HIV infection, the incidence was 9 cases per 1000 population, which is considerably higher than that in the general population [34].

The Miyazaki study revealed that the incidence of herpes zoster was higher in women than in men, and increased markedly after the age of 50 years in both sexes [3]. That study also revealed that the incidence varied according to the season, being higher in the summer than in the winter. Since the introduction of routine varicella immunization in Japan in 2014, the seasonal effect of herpes zoster has become less apparent [35].

## 4. Herpes Zoster Vaccines

Globally, two vaccines are available for herpes zoster. The first is based on the Oka strain of VZV, and vaccination with this strain has been shown to decrease the incidence of herpes zoster and postherpetic neuralgia [36]. In the United States, the Oka strain was used to develop the herpes zoster vaccine, Zostavax (Merck & Co., Inc., Kenilworth, NJ, USA). The vaccine was approved for use in adults over the age of 60 years to prevent herpes zoster and its complications in 2006, and it was subsequently recommended by ACIP in 2008 [37].

The second vaccine is a recombinant subunit vaccine containing the VZV glycoprotein E and the AS01B adjuvant system. The vaccine is called HZ/su (Shingrix, GlaxoSmithKline Biologicals, Rixensart, Belgium), and it is effective in preferentially reducing the risk of herpes zoster in adults over the age of 50 years [38]. In 2017, the HZ/su vaccine was approved in the United States for use in individuals over the age of 50 years to prevent herpes zoster. The vaccine requires two 0.5-mL doses, which are administered intramuscularly with a 2–6-month interval between doses [39]. The vaccine is recommended by ACIP for use in immunocompetent adults over the age of 50 years [25].

In 2016, the use of the freeze-dried Biken live attenuated varicella-zoster vaccine (BIKEN Co. Ltd., Osaka, Japan) was approved in Japan for the prevention of herpes zoster in adults over the age of 50 years. The Biken vaccine is essentially the same as Zostavax. The Shingrix vaccine was also approved in Japan in 2018 and became commercially available in 2020. In Japan, it is also indicated for the prevention of herpes zoster in adults over the age of 50 years.

In the United States, zoster vaccination coverage increased from 6.7% in 2008 to 34.5% in 2018 [40]. Safety concerns limit the use of live vaccines in immunocompromised patients. However, the recombinant zoster vaccine has been confirmed to be safe and immunogenic in certain immunocompromised patient groups [41]. Therefore, it can be used to vaccinate immunocompromised patients for whom a live vaccine is not an option. Further epidemiological studies are needed to confirm the efficacy of the recombinant zoster vaccine.

## 5. Association between the Epidemiology and Immunology of Varicella and Herpes Zoster

Humoral immunity plays a critical role in varicella development. In contrast, a decrease in VZV-specific CMI is thought to contribute to the development of herpes zoster [4]. In other words, while humoral immunity (antibody titer) persists after varicella infection or vaccination, CMI is thought to become compromised with age [4].

Immune boosting is thought to occur endogenously, i.e., subclinical reactivation or development of shingles, and exogenously, i.e., exposure to patients with VZV [29]. The epidemiological frequency of endogenous boosting is not well-understood, and exogenous boosting may be reduced by increasing vaccination coverage. The higher the incidence of varicella, the greater the chance that an individual who has previously had varicella will come in contact with individuals with varicella. Each contact can activate CMI against VZV and have a booster effect, thus sustaining immunity over a longer period. However, with the varicella vaccine becoming part of routine vaccination programs, the number of cases of varicella has decreased and the chance of coming into contact with individuals with varicella has decreased. As such, there are fewer opportunities for individuals to activate their CMI through the booster effect, and this change is likely to have contributed to the increase in the incidence of herpes zoster [35].

Both herpes zoster vaccines that are currently in use are effective at boosting CMI against VZV and preventing herpes zoster [8,42]. A simple method of measurement of CMI is needed to help identify those who need to be vaccinated.

## 6. Methods for Assessing Immunity to Varicella Zoster Virus

In the United States, the following are considered evidence of immunity [43]: (1) United States-born before 1980 (except for pregnant women and health care personnel); (2) documentation of two doses of varicella-containing vaccine at least 4 weeks apart; (3) diagnosis or verification of a history of varicella or herpes zoster by a health care provider; or (4) laboratory evidence of immunity or disease. For laboratory evidence, commercial assays are recommended to evaluate the level of disease-induced immunity; however, they are not recommended to evaluate the level of vaccine-induced immunity because of their low sensitivity [2,44]. In Japan, varicella vaccination coverage prior to the start of routine immunization in 2014 was only 30–40%, and individuals only received one dose of the vaccine [18]. As such, in Japan, antibody testing is typically used to evaluate the level of immunity to varicella.

Immunity against varicella is evaluated based on humoral immunity (antibody testing). Methods to evaluate the level of humoral immunity include the fluorescent-antibody-to-membrane-antigen (FAMA) test, immune adherence hemagglutination assay (IAHA), and enzyme-linked immunosorbent assay (ELISA). Among them, FAMA and glycoprotein-based ELISA (gpELISA) are preferred for evaluating vaccine-induced immunity because of their high sensitivity [45]. A World Health Organization position paper [46] also endorses the use of gpELISA for assessing the protective antibody level. While gpELISA kits are commercially available, they are not widely used in Japan, and a standard ELISA (Denka Seiken Co., Tokyo, Japan) is often used. The gpELISA and the Denka Seiken ELISA have been shown to have equivalent sensitivity to the FAMA test [47]. Thus, ELISAs are effective tools for evaluating the level of humoral immunity against VZV.

CMI is considered important in evaluating immunity against shingles [7]. Intradermal testing [8] is the only method that is used clinically to evaluate the level of CMI against VZV. Although other methods that measure the level of interferon gamma (IFN-γ), such as enzyme-linked immune absorbent spot (ELISPOT) [9] and IFN-γ release assay (IGRA) [10], are also available, these methods are not used in routine clinical practice. Methods used to evaluate CMI in tuberculosis include QuantiFERON-TB (QFT) (Quiagen, Hiden, Germany) and T-spot TB (T-spot) (Oxford Immunotec, Abingdon, United Kingdom) [48]. QFT is an ELISA-based method, and ELISPOT is used for T-spot. A comparative study demonstrated that QFT is more sensitive than T-spot [49]. An IGRA for VZV is based on the same method as QFT and does not require a cell isolation step after blood collection, making sample handling easier than with ELISPOT. Thus, ELISA is likely to be suitable for evaluating the level of CMI in clinical practice.

## 7. Conclusions

In the United States, many states and jurisdictions have achieved high immunization rates and require children to receive vaccinations for vaccine-preventable diseases before they start school [50,51]. In Japan, the varicella vaccine became part of routine immunization programs in 2014 [52]. However, routine immunization is not a requirement before starting school, and as a result, the immunization rate remains lower in Japan than in the United States. The basic reproduction number of varicella is thought to range from 8 to 10 [53], indicating that the current immunization coverage in Japan is insufficient to prevent varicella, and that additional efforts are needed to promote vaccination against varicella. In addition, while the incidence of herpes zoster is higher in individuals over the age of 50 years [3], the incidence will likely increase in younger individuals as the incidence of varicella decreases. Thus, vaccination against herpes zoster is needed for individuals with decreased CMI against VZV. In order to identify vulnerable individuals, it is important to develop a method to assess the activity of CMI.

## Data Availability

Not applicable.
